# LncRNA HAND2-AS1 inhibits proliferation and promotes apoptosis of non-small cell lung cancer cells by inactivating PI3K/Akt pathway

**DOI:** 10.1042/BSR20201870

**Published:** 2020-11-02

**Authors:** Ting Gao, Xiaoqiang Dai, Yindi Jiang, Xiaopeng He, Shuli Yuan, Peiwen Zhao

**Affiliations:** 1 Department of Respiratory Medicine, XianYang Central Hospital, Xianyang City, Shaanxi Province, 712000, P.R. China; 2 Internal Medicine-Oncology, XianYang Central Hospital, Xianyang City, Shaanxi Province, 712000, P.R. China; 3 Department of Blood Medicine, Xianyang City Central Hospital, 712000, P.R. China

**Keywords:** lncRNA HAND2-AS1, non-small cell lung cancer, PI3K/Akt pathway

## Abstract

Background: Non-small cell lung cancer (NSCLC) is a major subtype of lung cancer and is correlated with high incidence and mortality rate. Functionality of lncRNA HAND2-AS1 is only reported in endometrioid endometrial carcinoma and osteosarcoma. In our study, the role of HAND2-AS1 in NSCLC was investigated.

Methods: We first detected the expression of HAND2-AS1 in lung tissues and serum of both NSCLC patients and healthy controls by qRT-PCR. Correlation between HAND2-AS1 expression level and clinical data of NSCLC patients was analyzed by Chi-square test. NSCLC cells, and cell proliferation, cell apoptosis and expression of PI3K/Akt pathway-related proteins were detected by CCK-8 assay, cell apoptosis assay and Western blot, respectively.

Results: HAND2-AS1 expression was significantly down-regulated in NSCLC. HAND2-AS1 and tumor size of NSCLC patients were closely associated. Serum HAND2-AS1 can be used to effectively distinguish osteosarcoma patients from healthy controls, and it can also be used to predict prognosis of osteosarcoma patients. HAND2-AS1 overexpression inhibited osteosarcoma cell proliferation, promoted cell apoptosis, and down-regulated phosphorylation of PI3K/Akt pathway-related proteins. PI3K/Akt pathway inhibitor showed no significant effects on HAND2-AS1 expression, but reduced its effects on cell proliferation and apoptosis.

Conclusion: We conclude that HAND2-AS1 may suppress the proliferation of NSCLC cells by targeting PI3K/Akt pathway.

## Background

Lung cancer is a common and deadly malignancy that causes unacceptable high mortality rate [1,2]. In recent years, incidence of lung cancer shows an increasing trend, especially in developing countries such as China, aggregated environmental pollution and tobacco consumption is predicted to further increase the burden of lung cancer in near future [3]. As the major type of lung cancer, NSCLC accounts for more than 85% of all cases of lung cancer [4]. Onset and development of NSCLC lack typical clinical manifestations, and most patients were diagnosed at advanced stages with the existing of tumor distant metastasis [5], leading to poor survival. Therefore, in-time diagnosis is particularly critical.

PI3K/Akt pathway plays pivotal roles in many important aspects of human malignancies including NSCLC [6,7]. PI3K/Akt is the major cell survival pathway in cancers and inhibition of PI3K/Akt signaling provides a new way for cancer therapy [6,7]. Long (>200 nt) non-coding RNAs (lncRNAs) are non-coding RNA transcripts that participate in diverse pathological and physiological processes mainly by regulating related gene expression at multiple levels [8]. It has been well established that certain lncRNAs interact with PI3K/Akt pathway to perform their roles in cancer [9,10]. HAND2-AS1 is a newly identified lncRNA with characterized functionality only in osteosarcoma [11] and endometrioid endometrial carcinoma [12]. In our study, we observed that HAND2-AS1 may inhibit the proliferation of NSCLC cells by targeting PI3K/Akt pathway.

## Materials and methods

### Specimens

This is a retrospective study. Adjacent healthy tissues, tumor tissues, and serum samples of 94 patients with NSCLC (gender: 59 males and 35 females; age: 28 to 70 years; mean: 46.1 ± 7.3 years) who were treated in XianYang Central Hospital from January 2011 to January 2013 were collected. Inclusion criteria: (1) new NSCLC cases; (2) signed informed consent; (3) patients with complete follow-up data after surgery. Exclusion criteria: (1) patients currently have or have a history of other types of malignancies; (2) patients with other types of lung diseases; (3) patients treated in our hospitals before admission; (4) patients died of other diseases or accidents during follow-up. At the same time, serum samples of 64 healthy people (gender: 40 males and 24 females; age: 30–72 years; mean age: 46.9 ± 8.3 years) who received routine physiological examinations in our hospital during the same time period were also obtained to serve as control group. Healthy controls also signed informed consent. Ethics committee of aforementioned hospital approved the present study.

### NSCLC cells and transfection

Our *in vitro* experiments included two NSCLC cell lines (human) NCI-H23 and NCI-H522 and one normal lung epithelial (human) cell line BEAS-2B (ATCC, U.S.A.). A mixture of 90% Eagle’s Minimum Essential Medium and 10% FBS (Sigma-Aldrich) was used to cultivate all cells in an incubator (37°C, 5% CO_2_). HAND2-AS1 expression vector was constructed using pIRSE2-EGFP vector (Clontech, U.S.A.). This HAND2-AS1 (10 nM) cells were counted and 4 × 10^5^ cells were transfected with 10 nM HAND2-AS1 expression vector or 10 nM empty vector (negative control, NC) through Lipofectamine 2000 (Thermo Fisher Scientific, Inc.) mediated transfection. Cells were harvested at 24 h post-transfections to perform subsequent experiments. Three independent replicates were set for each experiment.

### Cell proliferation assay

Cell suspension (4 × 10^4^ per ml) was prepared by mixing 4 × 10^3^ cells with 1 ml cell culture medium. Cells were cultivated in a 96-well plate with 100 μl cell suspension per well at 37°C with 5% CO_2_, and 10 μl of CCK-8 solution (Sigma-Aldrich) was added at 4 h before the termination of cell culture. After that, 10 μL DMSO was added and ODs were measured at 450 nm. Three independent replicates were set for each experiment.

### Cell apoptosis analysis

Cells with transfections were washed with PBS (ice-cold), and staining in dark with FITC labeled Annexin-V and PI (Sigma-Aldrich) was performed 15 min. After that, flow cytometry was used to separate apoptotic cells. The group with the highest apoptotic cell percentage was set to ‘100%’, and all other groups were normalized to this group. Three independent replicates were set for each experiment.

### Real-time quantitative PCR

About 4 × 10^5^ cells or 0.015 g tissue (ground in liquid nitrogen) was mixed with 1 ml Trizol reagent (Invitrogen, U.S.A.) to extract total RNA. Trizol was also used to extract RNA from serum samples. RNA samples (200 μl) were thawed on ice and were centrifuged for 10 min at 12,000 *g* 4°C, followed by the removal of cell debris. The serum samples were then transferred to 2 ml DNA-LoBind tubes (Eppendorf, Germany), followed by the addition of 1.2 ml Trizol for RNA isolation. Following DNase I digestion, reverse transcription was performed to obtain cDNA and qPCR mixtures were prepared using SYBR Green Master Mix (Bio-Rad, U.S.A.). Primers used in PCR reactions were: 5′-GGGTGTTTACGTAGACCAGAACC-3′ (forward) and 5′-CTTCCAAAAGCCTTCTGCCTTAG-3′ (reverse) for lncRNA HAND2-AS1; 5′-GACCTCTATGCCAACACAG-3′ (forward) and 5′-AGTACTTGCGCTCAGGAGG-3′ (reverse) for β-actin. Conditions of PCR were: 40 s at 95°C, followed by 40 cycles of 95°C for 10 s and 60°C for 45 s. 2^−ΔΔCT^ method was used to process data. Three technical replicates were set for each PCR reaction.

### Western-blot

A total of 4 × 10^5^ cells were mixed with 1 ml RIPA solution (Sangon) to extract protein. Following denaturing in boiled water for 5 min, 10% SDS-PAGE gel was used to perform electrophoresis. PVDF membranes were used to perform gel transfer and blocking was performed for 2 h at room temperature in 5% non-fat milk. Blotting was performed using rabbit p-AKT (1:1200, ab18206, Abcam), AKT (1:1200, ab126811, Abcam), PI3K (1:1200, ab182651, Abcam), PI3K (1:1200, ab5451, Abcam), cytochrome *c* (1: 1200, ab90529, Abcam), and PI3K (1:1200, ab9485, Abcam) primary antibodies (overnight at 4°C) and goat IgG-HRP secondary antibody (1:800, MBS435036, MyBioSource). ECL (Sangon) was used to develop signal. Gray values were processed using Image J V1.6 software. Three independent replicates were set for each experiment.

### Statistical analysis

SPSS19.0 (SPSS Inc., U.S.A.) was used. Count data (age, gender, tumor size, tumor metastasis) were analyzed by Chi-squared test. Protein and mRNA expression data were expressed using mean values. Differences between two groups or among multiple groups were performed using unpaired *t* test or one-way ANOVA combined with Tukey test, respectively. *P*<0.05 was statistically significant.

## Results

### Expression of lncRNA HAND2-AS1 in tissues of 94 patients with NSCLC

**Figure 1:**
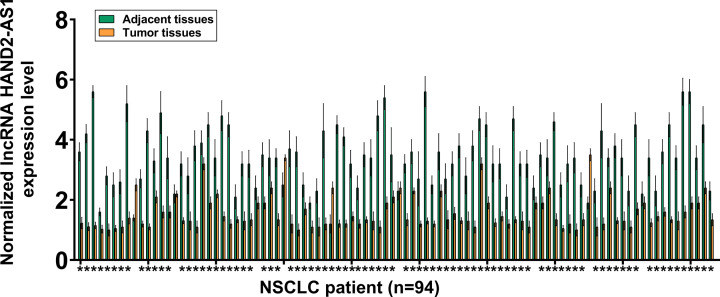
Expression of lncRNA HAND2-AS1 in tumor tissues, adjacent healthy tissues of 94 patients with NSCLC RT-qPCR was performed to determine the expression of HAND2-AS1 in paired tumor and adjacent healthy tissues from 94 NSCLC patients. PCRs were performed in three technical replicates and mean +/- SD values were presented. Notes: *, compared with adjacent heathy tissues, *P*<0.05.

QRT-PCR was performed to investigate the expression of HAND2-AS1 in tumor tissues, adjacent healthy tissues of 94 patients with NSCLC. As shown in Figure 1, in 85 out of 94 patients (90.4%), HAND2-AS1 expression level was lower in tumor tissues comparing to healthy tissues (*P*<0.05). Therefore, down-regulation of HAND2-AS1 may participate in NSCLC.

### Comparison of serum levels of HAND2-AS1 and the clinical values

**Figure 2:**
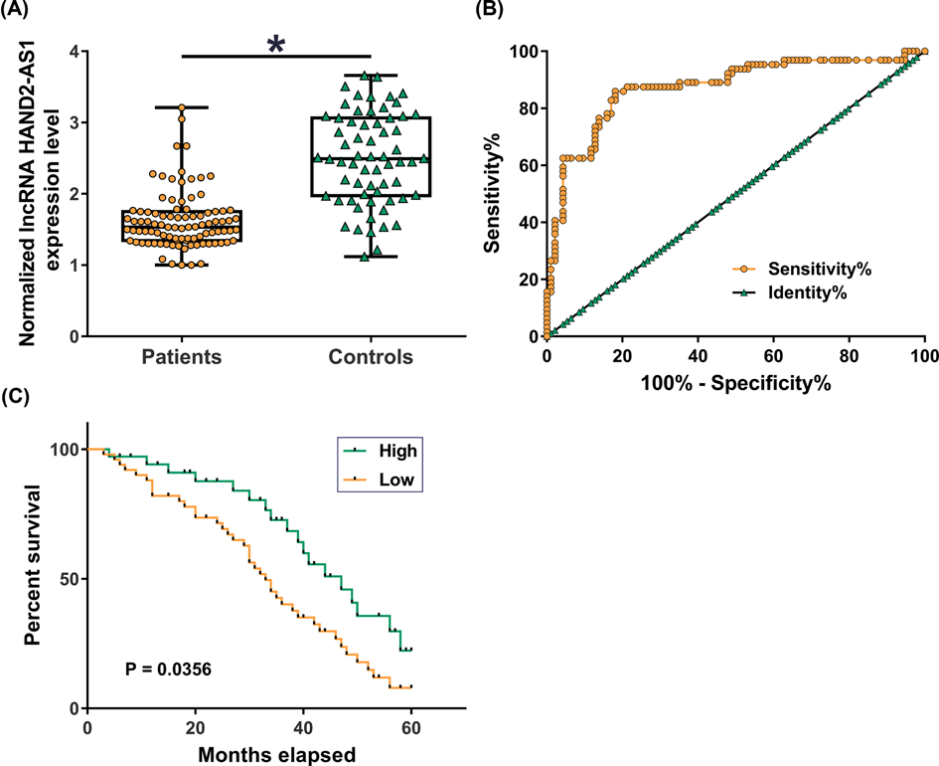
Comparison of serum levels of HAND2-AS1 and the diagnostic and prognostic values RT-qPCR was performed to determine the expression of HAND2-AS1 in plasma samples from 94 NSCLC patients and 64 healthy controls. PCRs were performed in three technical replicates and average values were presented (**A**). Diagnostic value of serum HAND2-AS1 for NSCLC analyzed by ROC curve analysis with NSCLC patients as true positive cases and healthy controls as true negative cases (**B**). NSCLC patients were divided into high (*n*=47) and low (*n*=47) HAND2-AS1 expression groups. Kaplan–Meier plotter was used to plot survival curves for both groups, followed by comparison by log-rank *t* test. (**C**) Notes:*, *P*<0.05.

Serum levels of HAND2-AS1 in NSCLC patients and the healthy control were measured using qRT-PCR. Unpaired *t* test analysis showed that serum HAND2-AS1 levels were significantly lower in NSCLC patients compared with control group (*P*<0.05). ROC curve was performed to analyze the values of HAND2-AS1 in NSCLC diagnosis. In this analysis, NSCLC patients were true positive cases and healthy controls were true negative cases. It was observed that area under the curve was 0.8734 (95% confidence interval: 0.8141–0.9328; standard error: 0.03027; Figure 2B, *P*<0.0001). To perform survival analysis, NSCLC patients were divided into high (*n*=47) and low (*n*=47) HAND2-AS1 expression groups. Kaplan–Meier plotter was used to plot survival curves for both groups, followed by comparison by log-rank *t* test. As shown in Figure 2C, overall survival of patients with low serum level of HAND2-AS1 was significantly worse than patients with high serum level of HAND2-AS1 (*P*<0.001). Therefore, plasma HAND2-AS1 may serve as a potential diagnostic and prognostic biomarker for NSCLC.

### Correlation between HAND2-AS1 and clinicopathological data of NSCLC patients

Chi square analysis was used to analyze the correlations between serum levels of HAND2-AS1 and clinicopathological data of NSCLC. Serum levels of HAND2-AS1 were not closely associated with patients’ gender and age, as well as distant tumor metastasis (Table 1). However, significant correlation was significantly correlated with tumor size.

**Table 1:** Correlation between serum levels of HAND2-AS1 and clinicopathological data of NSCLC patients

Items	Groups	Cases	High-expression	Low-expression	χ²	*P* value
Gender	Male	59	27	32	1.14	0.29
	Female	35	20	15		
Age	>45 (years)	48	22	26	0.68	0.41
	<45 (years)	46	25	21		
Primary tumor diameter	>7cm	28	10	18	4.37	0.11
	3–7 cm	48	25	23		
	<3 cm	18	12	6		
Distant tumor metastasis	Yes	45	20	25	1.07	0.30
	No	49	27	22		

### Effects of on HAND2-AS1 overexpression on PI3K/Akt pathway-related proteins

**Figure 3:**
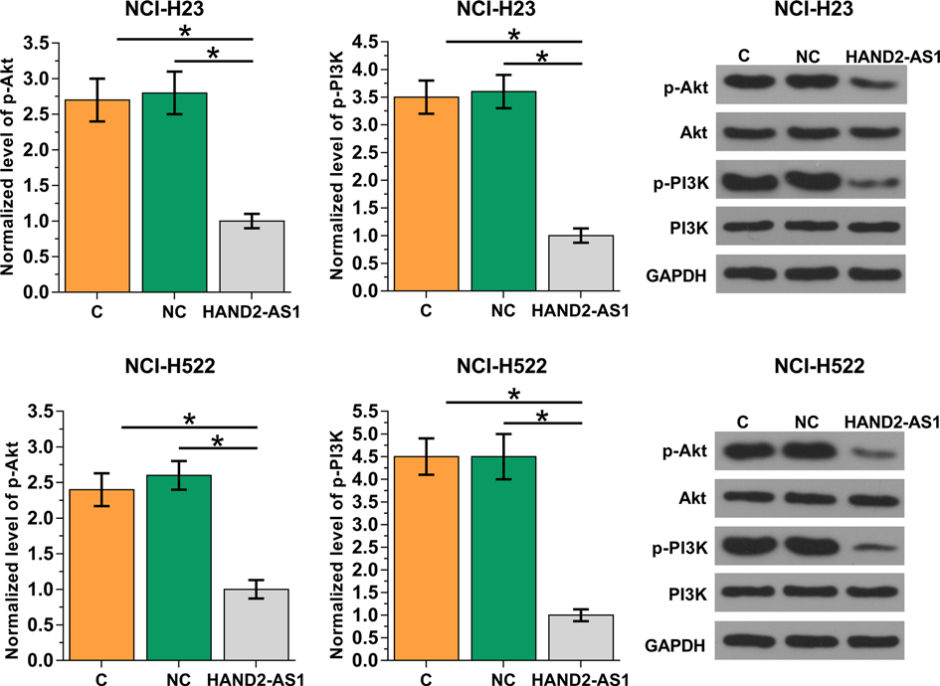
Effects of on HAND2-AS1 overexpression on PI3K/Akt pathway-related proteins HAND2-AS1 overexpression was achieved in both NCI-H23 and NCI-H522 cells, followed by Western blot to determine the expression levels of p-Akt, Akt, p-PI3K and PI3K. Experiments were repeated in three biological replicates and mean ± SD values were presented and compared. Notes: *P<0.05.

Data in Table 1 suggest that HAND2-AS1 is likely involved in the growth of NSCLC. PI3K/Akt pathway plays pivotal roles in cancer biology [6]. In the present study, HAND2-AS1 overexpression in cells of two NSCLC cell lines NCI-H23 and NCI-H522 and one normal lung tissue cell line BEAS-2B, and the effects of HAND2-AS1 overexpression on PI3K/Akt-related proteins were explored by Western blot. As shown in Figure 3, HAND2-AS1 overexpression did not significantly affect expression levels of PI3K and AKT, but significantly promoted the phosphorylation of PI3K and AKT in cells of two NSCLC cell lines NCI-H23 and NCI-H522, but not in cells of normal lung tissue cell lineBEAS-2B (data not shown). In contrast, treatment with PI3K activator SC79 (10 μM, Sigma-Aldrich) showed no significant effects on HAND2-AS1 (data not shown). Therefore, HAND2-AS1 may suppress PI3K/Akt to participate in NSCLC. See Supplementary File S1 for represent images of Western blot analysis.

### Effects of HAND2-AS1 overexpression and Akt inhibitor on cell behaviors

**Figure 4:**
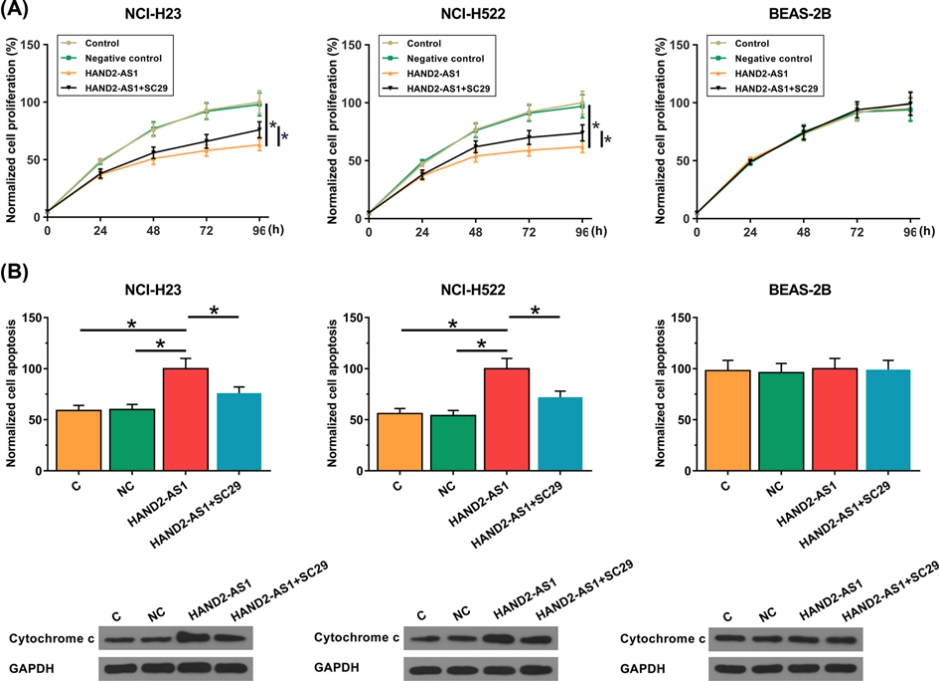
Effects of HAND2-AS1 overexpression and Akt inhibitor on cell proliferation and apoptosis CCK-8 assay and cell apoptosis assay was performed to detect cell the proliferation (**A**) and apoptosis (**B**) of two NSCLC cell lines NCI-H23 and NCI-H522, as well as one normal lung cell line BEAS. Western blot was performed to detecte the levels of cleaved cytochrome *c* in each group of the cell apoptosis assay, and consistent results were observed (B). Experiments were repeated in three biological replicates and mean ± SD values were presented and compared.

CCK-8 assay and cell apoptosis assay was performed to detect the proliferation and apoptosis of two NSCLC cell lines NCI-H23 and NCI-H522, as well as one normal lung cell line BEAS. As shown in Figure 4, HAND2-AS1 overexpression significant inhibited proliferation (Figure 4A) and promoted apoptosis (Figure 4B) of cells of two NSCLC cell lines NCI-H23 and NCI-H522 but not cells of normal lung tissue cell line BEAS-2B. In addition, treatment with PI3K activator SC79 (10 μM) led to reduced effects of HAND2-AS1 overexpression significant on cell proliferation (Figure 4A) and apoptosis (Figure 4B). Western blot was performed to detect the levels of cytochrome *c* in each group of the cell apoptosis assay, and consistent results were observed (Figure 4B). The data suggested that HAND2-AS1 may suppress the proliferation of NSCLC cells, and promote the apoptosis of NSCLC cells through the inhibition of PI3K/Akt signaling. See Supplemental File S1 for represent images of Western blot analysis.

## Discussion

The key finding of our study is that HAND2-AS1 as a tumor suppressor lncRNA in osteosarcoma [11] and endometrioid endometrial carcinoma [12] also regulated the behaviors NSCLC cells. The action of HAND2-AS1 in NSCLC is very likely achieved by inactivation of PI3K/Akt pathway.

The involvement of lncRNAs in NSCLC has been extensively reported by previous studies. Different lncRNAs may have different expression patterns and play different roles in different types of malignancies. LncRNA-UCA1 and SNHG1 were significantly up-regulated in NSCLC tissues than in paired adjacent healthy tissues, and the up-regulation of UCA1 and SNHG1 promotes tumor progression [13,14]. In contrast, lncRNA MEG3 was down-regulated in NSCLC, and MEG3 overexpression inhibits the growth of tumor [15]. Down-regulation of HAND2-AS1 was observed in osteosarcoma [11] and endometrioid endometrial carcinoma [12]. In our study, most patients with NSCLC showed a significantly decreased expression level of HAND2-AS1 in tumor tissues than in adjacent healthy tissues. Those data indicate that HAND2-AS1 may also play a role as tumor suppressor lncRNA in NSCLC.

Early diagnosis of NSCLC before the occurrence of distant tumor metastasis is the key for patients’ survival. The onset of human diseases is affected certain substances in blood [16]. In the present study, significantly increased serum levels of HAND2-AS1 were observed in NSCLC patients than in healthy controls. ROC curve analysis proved that serum HAND2-AS1 can be used to effectively distinguish NSCLC patients from healthy controls. In addition, low serum HAND2-AS1 level was proved to be significantly correlated with shorter survival time after surgery. Therefore, serum NSCLC may serve as a potential diagnostic and prognostic biomarker for the diagnosis of NSCLC. However, down-regulation of HAND2-AS1 is not specific to NSCLC [11,12]. Therefore, multiple markers should be combined in the diagnosis and prognosis to improve the accuracy.

Our study also observed that serum levels of HAND2-AS1 were significantly correlated with tumor size but not distant tumor metastasis. A recent study has proved that HAND2-AS1 is involved in the regulation of invasion and metastasis in endometrioid endometrial carcinoma [12], indicating that endometrioid endometrial carcinoma and NSCLC may have different pathogenesis and an lncRNA may play different roles in different types of malignancies. Activation of PI3K/Akt pathway promotes tumor growth in a variety type of malignancies [17,18], while inactivation of PI3K/Akt pathway inhibit tumor growth [19]. In our study, HAND2-AS1 overexpression significantly promoted the activation of PI3K/Akt pathway, and inhibited the proliferation and promoted the apoptosis of NSCLC cells. In addition, Akt activator showed no significant effect on HAND2-AS1 expression but significantly reduced the effects of HAND2-AS1 overexpression on NSCLC cell proliferation and apoptosis. Those data suggest that HAND2-AS1 may inhibit the proliferation of NSCLC cells by targeting PI3K/Akt pathway.

It is also worth to note that HAND2-AS1 overexpression shown no significant effects on proliferation and apoptosis of normal human lung cells and the activation of PI3K/Akt pathway in those cells. Therefore, HAND2-AS1 may serve as a safe therapeutic target for NSCLC.

In conclusion, HAND2-AS1 expression was significantly down-regulated in NSCLC, and reduced serum level of HAND2-AS1 may assist the diagnosis and prognosis of NSCLC. HAND2-AS1 overexpression significantly promoted the activation of PI3K/Akt pathway, and inhibited the proliferation and promoted the apoptosis of NSCLC cells. We conclude that HAND2-AS1 may inhibit the proliferation of NSCLC cells by targeting PI3K/Akt pathway.

## Data Availability

The analyzed data sets generated during the study are available from the corresponding author on reasonable request.

## Abbreviations

lncRNA: non-coding RNA
NSCLC: non-small cell lung cancer

## References

[1] Ma J., Ward E.M., Smith R. et al. (2013) Annual number of lung cancer deaths potentially avertable by screening in the United States. Cancer 119, 1381–1385 10.1002/cncr.27813 23440730

[2] Torre L.A., Siegel R.L. and Jemal A. (2016) Lung cancer statistics. Lung cancer and personalized medicine, pp. 1–19, Springer, Cham 10.1007/978-3-319-24223-1_1 26667336

[3] Chen W., Zheng R., Zeng H. et al. (2015) Annual report on status of cancer in China, 2011. Chin. J. Cancer Res. 27, 2–12 10.1186/s40880-015-0001-2 25717220 PMC4329176

[4] Ettinger D.S., Akerley W., Bepler G. et al. (2010) Non–small cell lung cancer. J. Natl. Compr. Canc. Netw. 8, 740–801 10.6004/jnccn.2010.0056 20679538

[5] Rosen L.S., Gordon D., Tchekmedyian S. et al. (2003) Zoledronic acid versus placebo in the treatment of skeletal metastases in patients with lung cancer and other solid tumors: a phase III, double-blind, randomized trial-the Zoledronic Acid Lung Cancer and Other Solid Tumors Study Group. J. Clin. Oncol. 21, 3150–3157 10.1200/JCO.2003.04.105 12915606

[6] Mayer I.A. and Arteaga C.L. (2016) The PI3K/AKT pathway as a target for cancer treatment. Annu. Rev. Med. 67, 11–28 10.1146/annurev-med-062913-051343 26473415

[7] Fumarola C., Bonelli M.A., Petronini P.G. et al. (2014) Targeting PI3K/AKT/mTOR pathway in non small cell lung cancer. Biochem. Pharmacol. 90, 197–207 10.1016/j.bcp.2014.05.011 24863259

[8] Fatica A. and Bozzoni I. (2014) Long non-coding RNAs: new players in cell differentiation and development. Nat. Rev. Genet. 15, 7–21 10.1038/nrg3606 24296535

[9] Cai Q., Wang Z.Q., Wang S.H. et al. (2016) Upregulation of long non-coding RNA LINC00152 by SP1 contributes to gallbladder cancer cell growth and tumor metastasis via PI3K/AKT pathway. Am. J. Transl. Res. 8, 4068–4081 27829993 PMC5095302

[10] Shen S., Liu H., Wang Y. et al. (2016) Long non-coding RNA CRNDE promotes gallbladder carcinoma carcinogenesis and as a scaffold of DMBT1 and C-IAP1 complexes to activating PI3K-AKT pathway. Oncotarget 7, 72833–72844 10.18632/oncotarget.12023 27637083 PMC5341947

[11] Kang Y., Zhu X., Xu Y. et al. (2018) Energy stress-induced lncRNA HAND2-AS1 represses HIF1α-mediated energy metabolism and inhibits osteosarcoma progression. Am J Cancer Res. 8, 526–537 29637006 PMC5883101

[12] Yang X., Wang C.C., Lee W.Y.W. et al. (2018) Long non-coding RNA HAND2-AS1 inhibits invasion and metastasis in endometrioid endometrial carcinoma through inactivating neuromedin U. Cancer Lett. 413, 23–34 10.1016/j.canlet.2017.10.028 29107108

[13] Nie W., Ge H., Yang X. et al. (2016) LncRNA-UCA1 exerts oncogenic functions in non-small cell lung cancer by targeting miR-193a-3p. Cancer Lett. 371, 99–106 10.1016/j.canlet.2015.11.024 26655272

[14] Cui Y., Zhang F., Zhu C. et al. (2017) Upregulated lncRNA SNHG1 contributes to progression of non-small cell lung cancer through inhibition of miR-101-3p and activation of Wnt/β-catenin signaling pathway. Oncotarget 8, 17785–17794 10.18632/oncotarget.14854 28147312 PMC5392286

[15] Lu K., Li W., Liu X. et al. (2013) Long non-coding RNA MEG3 inhibits NSCLC cells proliferation and induces apoptosis by affecting p53 expression. BMC Cancer 13, 461 10.1186/1471-2407-13-461 24098911 PMC3851462

[16] Weber D.G., Johnen G., Casjens S. et al. (2013) Evaluation of long noncoding RNA MALAT1 as a candidate blood-based biomarker for the diagnosis of non-small cell lung cancer. BMC Res. Notes 6, 518 10.1186/1756-0500-6-518 24313945 PMC4029199

[17] Dong Y., Liang G., Yuan B. et al. (2015) MALAT1 promotes the proliferation and metastasis of osteosarcoma cells by activating the PI3K/Akt pathway. Tumour Biol. 36, 1477–1486 10.1007/s13277-014-2631-4 25431257

[18] Fu Q.F., Liu Y., Fan Y. et al. (2015) Alpha-enolase promotes cell glycolysis, growth, migration, and invasion in non-small cell lung cancer through FAK-mediated PI3K/AKT pathway. J. Hematol. Oncol. 8, 22 10.1186/s13045-015-0117-5 25887760 PMC4359783

[19] Chen S., Fisher R.C., Signs S. et al. (2017) Inhibition of PI3K/Akt/mTOR signaling in PI3KR2-overexpressing colon cancer stem cells reduces tumor growth due to apoptosis. Oncotarget 8, 50476–50488 10.18632/oncotarget.9919 28881576 PMC5584153

